# Next Meeting Texas State Medical Association

**Published:** 1886-02

**Authors:** 


### THE NEXT MEETING OF THE TEXAS STATE MEDICAL ASSOCIATION.

Everything indicates that the coming annual convention of this progressive society, which will be held in Dallas, beginning on Tuesday, April 26, and holding four days, will be the largest, most interesting, and most important ever yet held. The published Transactions for '85 have attracted such widespread attention, and excited such favorable comment at the hands of the entire medical press of the country, that Texas physicians who are not members of the organization have had their pride and ambition aroused, and many will attend this meeting for the first time; while the members, stimulated and encouraged by the flattering notices of the papers published in the Transactions, will strive to make even a better show. It is a fortunate coincidence that the State Pharmaceutical Association will meet at Dallas at the same time. This will attract a larger number of physicians than would otherwise attend; and also a larger attendance by agents, and display of chemicals from the houses of manufacturing chemists than otherwise. We have been notified by Messrs Park, Davis & Co., and by Mr. J. W. Lambert, of Listerine fame, that they will make exhibits. It is hoped all delinquent members will attend and be reinstated, which, it is understood, they can do by paying only the membership fee—back dues being remitted. The social feature of the occasion will be grand, as we learn that extensive and elaborate preparations are being made to entertain the large number of visitors; and the ladies, God bless them, are actively aiding the committee.

